# Photothermal-Based Multiplex Nested Digital PCR System for Rapid Detection of Foodborne Pathogens

**DOI:** 10.3390/mi15040435

**Published:** 2024-03-25

**Authors:** Junwei Li, Xinyi Liang, Jinsong Ma, Jianye Cheng, Hui Wang, Xuzhao Wang, Jie Jayne Wu, Hailong An

**Affiliations:** 1 Institute of Biophysics, School of Health Sciences and Biomedical Engineering, Hebei University of Technology, Tianjin 300401, China; junwei_li@hebut.edu.cn (J.L.); wanghuihebut@163.com (H.W.);; 2 School of Science, Hebei University of Technology, Tianjin 300401, China; 3 School of Mechanical Engineering, Hebei University of Technology, Tianjin 300401, China220438@stu.hebut.edu.cn (J.C.); 4 Department of Electrical Engineering and Computer Science, The University of Tennessee, Knoxville, TN 37996, USA

**Keywords:** photothermal effect, multiplex, digital PCR, foodborne pathogens

## Abstract

The rapid and sensitive detection of foodborne pathogens is crucial for ensuring food safety. Among virus testing methods, polymerase chain reaction (PCR) has served as the gold-standard technique in most food safety regulation organizations. However, to enhance the speed and efficiency of PCR, novel approaches are continually being explored. In this work, leveraging the photothermal effects and high thermal conductivity of gold nanoparticles, we have significantly improved the heating and cooling rates of thermal cycles, enabling ultra-fast PCR detection. Specifically, we present a pre-degassing multiplex digital PCR chip integrated with gold nanoparticles. We further developed a portable system with a light source for photothermal heating cycling, along with an optoelectronic sensor to analyze PCR amplification products after rapid thermal cycling. As proof of concept, the proposed chip and portable device was applied for the on-site detection of several types of foodborne pathogens, including Escherichia coli, Listeria monocytogenes, Staphylococcus aureus, and *Salmonella*. The whole system could distinguish those pathogens within 20 min, showing good potential for the rapid detection of multiple types of foodborne pathogens.

## 1. Introduction

The detection of multiple types of foodborne pathogens is of paramount importance in food safety research and applications [1]. Polymerase chain reaction (PCR) is widely recognized as the gold standard for identifying pathogen species [2] due to its exceptional sensitivity and the capability for multiplexing. In the recent decades, significant progress has been made in the development and commercialization of PCR equipment [3] for the detection of foodborne pathogens. Among these approaches, digital polymerase chain reaction (dPCR) [4] using micro-reactors emerged as a promising approach to detect various foodborne pathogens [5,6], e.g., Escherichia coli, Listeria monocytogenes, Staphylococcus aureus, and *Salmonella*.

With high sensitivity and absolute quantities analysis ability, dPCR technology enables the ultra-high sensitivity down to the single-molecule level [7]. In dPCR devices, the PCR reaction liquid samples are partitioned into tens of thousands of micro chambers or droplets [8], with most compartments containing zero or one target DNA molecule. Due to the small volume of those chambers or droplets [9], as well as the Poisson distribution principles, the dPCR allows the direct monitoring of each single target DNA in a highly sensitive and quantitative way [10]. In our previous works [11,12] for meat adulteration detection applications, we developed a 40 × 40 chamber-based digital PCR microfluidic device that is compatible with fluorescence image read-out systems and removes bubbles via a pre-degassing operation.

Despite advancements in micro chamber array and droplet microfluidic technologies, conducting nucleic acid amplification thermal cycling in an ultra-fast manner remains a challenge. Various microfluidic PCR systems have thus far been developed to speed up the heating or cooling process in the PCR tests. Capitalizing on the photothermal effect, Luke Lee’s group demonstrated an ultra-fast photonic PCR method using the plasmonic photothermal effect [13]. The plasmon-excited gold film enables a rapid heating rate as high as 12.79 ± 0.93 °C/s for PCR tests. Kang et al. reported a single-shot multi-channel plasmonic real-time PCR technology [14] for multi-target detection.

Recently, Jia’s group developed a sub-five-minute ultra-fast electrowetting-on-dielectric (EWOD) PCR approach [15] using digital microfluidics. They employed an aqueous thermometer to enable accurate in-droplet thermal regulation for efficient ultrafast PCR within 5 min. Since the droplet size is sub-microliter scale, the EWOD-based PCR works well for low-concentration DNA targets. However, at higher DNA target concentrations, it may contain more-than-one target DNA in one single droplet, reducing the sensitivity of dPCR method. 

In this work, we aim to develop a photothermal heating multiplex chamber-based digital PCR (cdPCR) microfluidic method for foodborne pathogen detection. The conceptional cdPCR system demonstrates the full functions of PCR reagents’ loading and self-digitalization, a photothermal heating module for nucleic acid amplification, multiplex fluorescence imaging at four channels, and image analysis to read the numbers of positive chambers for each type of foodborne pathogen.

## 2. Materials and Methods

### 2.1. System Description and Working Principles

**Figure 1:**
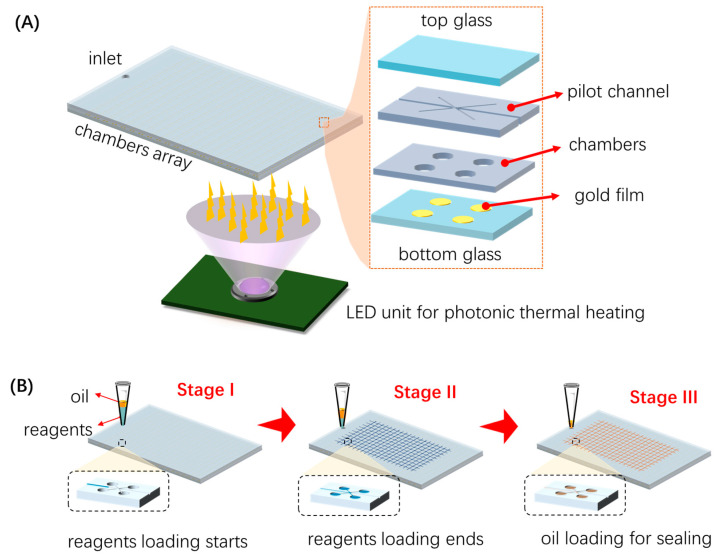
Schematic of the photothermal heating digital PCR system: (**A**) the structure of the self-digitization chip with a blind outlet and an external LED heating system; (**B**) the flow chart of the liquid loading process.

As shown in Figure 1A, the photothermal heating digital PCR system consisted of a microfluidic chamber array with an inlet and an LED array for the photothermal heating module. The microfluidic chip was pre-degassed for sample loading by negative pressure within the porous PDMS (Polydimethylsiloxane) block. The chambers array has 100 × 100 (the chamber density is improved compared with our previous work [11] on 40 × 40 chambers) micro chambers (100 microns in diameter and 20 microns in height). The chamber array consists of a pilot channel layer and a chamber layer. It was fabricated via standard soft lithography technology; more details can be found in our previous work [11]. The advantage of the updated design is the improvement of the sensitivity of digital PCR by the increasing of number of chambers. On the other hand, it would take a longer time to perform a satisfied self-digitization if we used the same channel height as before. Thus, we modified the previous design and fabricated the pre-degassed device with a chamber height of 20 microns only. 

Another new feature of the proposed design is that, in this work, a patterned gold film was deposited on the thin bottom glass (500 microns in thickness). A significant increase in the optical absorption could be attributed to the plasmonic resonance photothermal effect of the gold film. Thus, the thermal cycles could be performed via photothermal heating and nature cooling. Here, the LED module was powered and controlled by a PWM controller using STM 32 microcontroller. The low-cost and portable design made the system an ideal PCR heating source for nucleic acid amplification. 

As illustrated Figure 1B, the PCR reagents’ loading and self-digitalization were performed in three stages. Before the sample loading operations, the PCR reagent was preloaded in a pipette tip. Meanwhile, the PDMS chip was pre-degassed in a vacuum box (Fujiwara PC-3, Taizhou, China) for no less than 30 min under a pressure of around −1 kPa. Due to the air permittivity and solubility of the porous PDMS material, the pre-degassed microfluidic chip was able to provide negative pressure and allowed us to pump the PCR reagent from the inlet. To avoid any potential evaporation or aerosol contamination, oil was also preloaded upon the top surface of the PCR reagents.

### 2.2. PCR Experiments Design

All the pathogens were sourced from National Center for Veterinary Culture Collection of China, and their DNA were extracted using commercial kit from TransGen, Beijing, China. The initial concentrations were measured using a Multiskan SkyHigh ultraviolet spectrophotometer (Thermal Fisher Scientific, Waltham, MA, USA ), and then they were diluted into 1200 copies/μL. Food samples (beef) were purchased from local market; raw milk was purchased from a local dairy farm. Beef was grinded into powder and cultured by following the China national standards for pathogen culturing protocols. Gold nanoparticles were added into the DNA template reagent to improve the thermal conductivity of the PCR reagents.

### 2.3. Primer Design and Data Processing Method

To realize the multiplexing detection of foodborne pathogens, four kinds of fluorescence channel were used in this work. As shown in Table 1, the primers of four kinds of foodborne pathogens were designed using Primer 5.0 software. Here, all the primers had a general primer sequence of Gexp (forward: AGGTGACACTAGAATA; and Reverse: GTACGACTCACTATAGGGA). The sizes of these target gens were as the follow: *E. coli* (139 bp), *L. monocytogenes* (235 bp), *S. aureus* (316 bp), and *Salmonella* (158 bp). 

Their probes were detected under HEX (hexachlorocyclohexane, 515~535 nm/560~580 nm), FAM (6-carboxyfluorescein, 450~490 nm/510~530 nm), ROX (X-rhodamine, 557~587 nm/587~607 nm), and CY5 (cyanine dyes 5, 620~650 nm/675~690 nm) fluorescent channels, respectively. 

As the thermal cycling finished, the fluorescence images of the multiplexing dPCR chip were optically observed via an inversed fluorescence microscope (Nikon Eclipse Ti-S, Tokyo, Japan) equipped with a CCD (Charge-Coupled Device) camera (Nikon DS-Qi2, Tokyo, Japan). These fluorescence images of each pathogen were further analyzed using ImageJ2 software. The number of positive chambers was read out based on a threshold value of the fluorescence intensity of each chamber. Then, the target DNA concentration was calculated via this Poisson distribution equation [16]:

$$N=\frac{\lambda }{v}=\frac{-ln(1-N_{pos}/n)}{\pi D^{2}h/4}$$

where N is the calculated copy numbers of target DNA, $N_{pos}$ is the read-out numbers of positive chambers from the experimental fluorescence images, n is the total numbers of micro chambers (here n = 10,000), and D,h are the diameter and height of the micro chamber (here *D* = 100 μm and *h* = 20 μm).

## 3. Results and Discussions

### 3.1. Temperature Profiles

**Figure 2:**
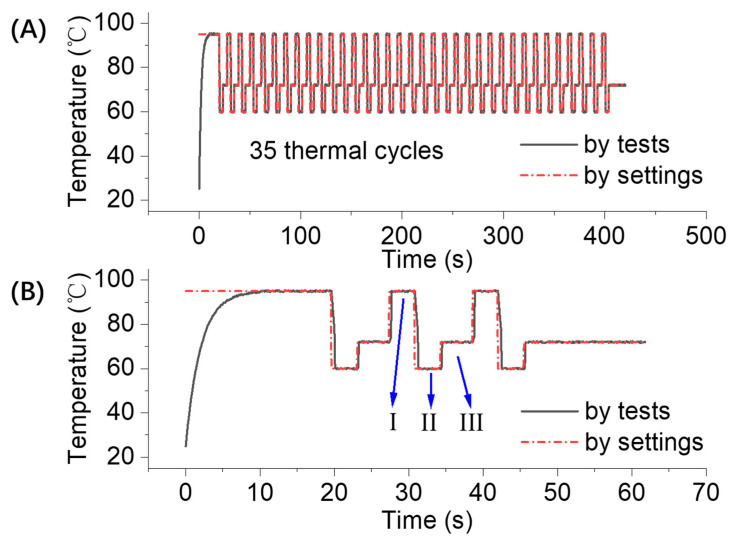
The photothermal heating/cooling process with light on/off: (**A**) the temperature profile as a function of time for 35 thermal cycles in all; (**B**) the temperature profile details showing the heating/cooling performance in the PCR process. The three phases labeled with I, II, and III stands for the typical temperature profile in one thermal cycle, as the following: I (95 °C denaturation for about 4.7 s), II (60 °C denaturation for about 4.5 s), and III (72 °C denaturation for about 4.6 s).

To show the performances of the heating and cooling rates of photothermal cycles, Figure 2 shows the photothermal heating/cooling process with the LED light on/off. In this work, complete PCR thermal cycling consists of three typical temperatures, that is, 95 °C denaturation for 5 s, 60 °C annealing for 5 s, and 72 °C extension for 5 s. As demonstrated, Figure 2B shows the temperature profiles labeled with I (95 °C denaturation for about 4.7 s), II (60 °C denaturation for about 4.5 s), and III (72 °C denaturation for about 4.6 s). The experimental temperature curve matches well with the expected profile.

Figure 2A shows the temperature profile of 35 ultra-fast photothermal heating/cooling cycles within 7 min. Here, we define the time-average heating/cooling rate as the value calculated by measuring the temperature difference between successive temperature maxima and minima and then dividing it by their time intervals. From the experimental data determined via tests, the heating and cooling rates of our system were around 10 °C/s and 8 °C/s, respectively. In general, temperature control is the critical factor in PCR for efficient DNA amplification. Thus, most commercial PCR thermal cyclers use the thermoelectric cooler (TEC) component to perform fast heating and cooling thermal cycling. For example, as reported in Ref. [17], the heating and cooling rates of the TEC Peltier element were about 8.3 °C/s and 8 °C/s, respectively. Recently, Ling et al. [18] proposed a gradual thermal conductivity design to increase the temperature uniformity, and they reached average heating/cooling rates of 5.52 and 5.28 °C/s, respectively. Demonstrating the limits of this method, their heating/cooling rates were around 7.2 and 6.12 °C/s, respectively. Compared with the heating and cooling rates of the PCR thermal cycler using the thermoelectric cooler effect or water-cooled or air-cooled effect [17,18] above, the device proposed in this work shows a significant improvement in both the heating and cooling processes. 

### 3.2. Verifications of the Primer Design and Thermal Cyclings

**Figure 3:**
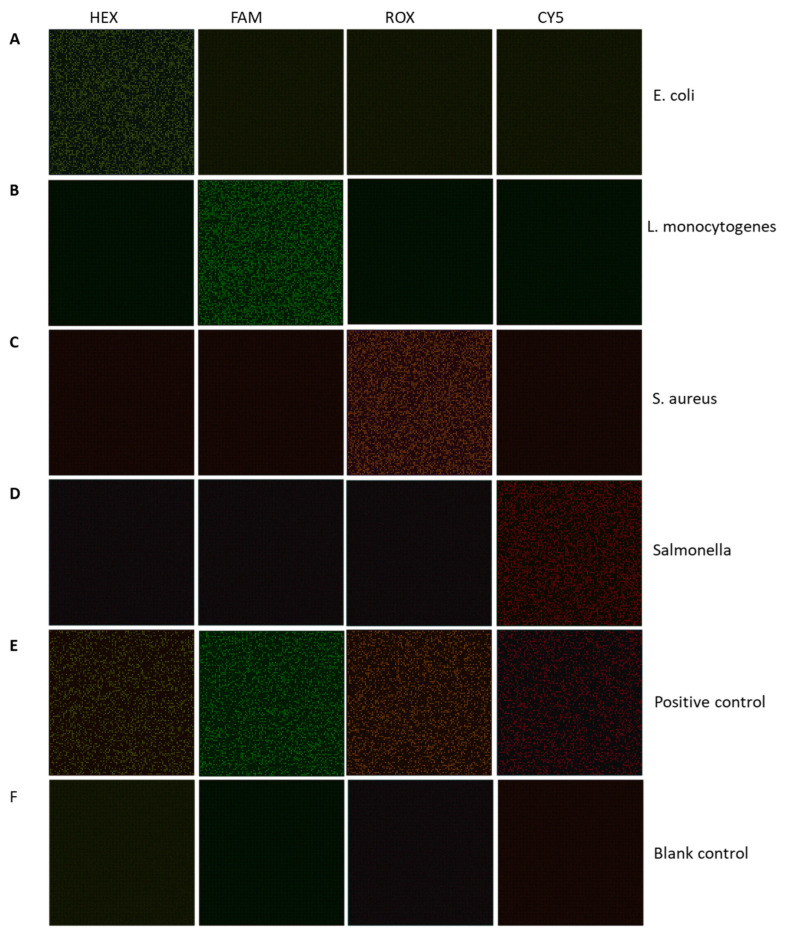
Experimental results of multiplexing dPCR to verify the primer design. From left to right are the fluorescence images from HEX, FAM, ROX, and CY5 channels, respectively. From top to bottom are the fluorescence images of target DNA templates from (**A**) *E. coli*, (**B**) *L. monocytogenes*, (**C**) *S. aureus*, (**D**) *Salmonella*, (**E**) positive control templates containing all four kinds of foodborne pathogens, and (**F**) blank control reagents using buffer only.

Concerning the effectiveness of the primer design for these foodborne pathogens, it is suggested to conduct a proof-of-concept test. As shown in Table 1, the four types of probes were designed as the indicator for the detection of specific primers of each kind of foodborne pathogen. To verify the performance of the primers design, a pre-test was carried out. As shown in Figure 3, a traditional PCR test of six samples was conducted by a commercial thermal heater (WH-TC-01 PCR temperature controller, Wenhao Co., Ltd., Suzhou, China) for the flat microfluidic chip.

In Figure 3, from left to right are the fluorescence images from the HEX, FAM, ROX, and CY5 channels, respectively. From top to bottom are the fluorescence images of target DNA template from (A) *E. coli*, (B) *L. monocytogenes*, (C) *S. aureus*, (D) *Salmonella*, and (E) positive control templates containing all the four kinds of foodborne pathogens, as well as (F) blank control reagents using buffer only. It can be seen in Figure 3A that the *E. coli*-positive template is positive in the HEX channel only. Correspondingly, the *L. monocytogenes*-positive template is positive in the FAM channel only, the *S. aureus*-positive template is positive in the ROX channel only, and the *Salmonella*-positive template is positive in the CY5 channel only.

Since the gold nanoparticles were used to improve the thermal conductivity of the PCR mix, here, we also conducted a photothermal test to optimize the concentration of gold nanoparticles. Here, we conducted a uniform PWM (pulse width modulation) regulation with a duty cycle of 50% as the heating source. Then, shining light was applied onto the liquid reagents, which were heated or cooled with the same power. Since the photothermal heating efficiency was proportional to the optical absorption property of the nanoparticles, it was reasonable to optimize the concentration of nanoparticles. According to the real-time temperature profiles, it was found that the concentration of gold nanoparticles influenced the heating/cooling rate significantly. It was found that the optimized protocol used to make a mixture of gold nanoparticles (>0.75 A520) and PCR reagents was about 1:10 *v*/*v*. The reason why the nanoparticles could increase the photothermal heating efficacy may lie in the optical density of the nanoparticles. 

Moreover, the positive control templates containing all four kinds of foodborne pathogens show positive results in all four fluoresce channels. Otherwise, the blank control containing no pathogens shows negative results in all four fluoresce channels. Thus, it could be verified that the reagent loading, the primer and probe design, and thermal cycling settings were reasonable for the multiplexing of foodborne pathogen applications. 

### 3.3. Verifications of Quantitative Capability

**Figure 4:**
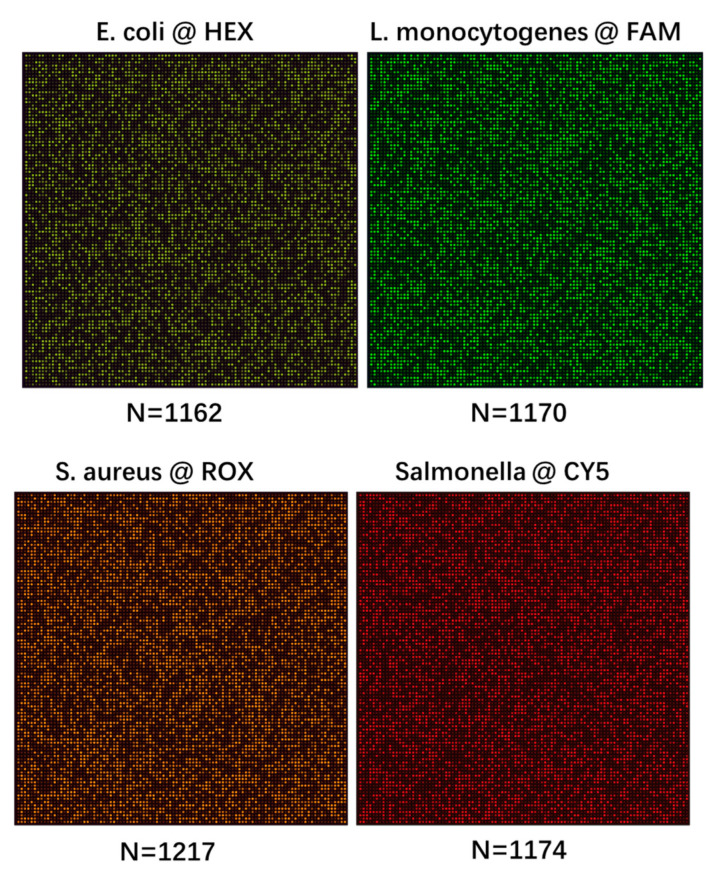
Experimental results of four kinds of foodborne pathogens and four fluorescence channels with DNA concentrations of ~1200 copies/μL.

To verify the quantitative analysis capability of our method, Figure 4 shows the experimental results of four kinds of foodborne pathogens, as well as four fluorescence channels with DNA concentrations of ~1200 copies/μL. As shown in Figure 4, the positive chamber of each pathogen is 4600 for *E. coli*, 4622 for *L. monocytogenes*, 4754 for *S. aureus*, and 4634 for *Salmonella*. There are 100 × 100 chambers in the nested digital PCR chip; thus, the calculated target DNA numbers of the above pathogen templates are 1162 copies/μL, 1170 copies/μL, 1217 copies/μL, and 1174 copies/μL, respectively. The experimental results verify the quantitative capability of the nested digital PCR chip.

**Figure 5:**
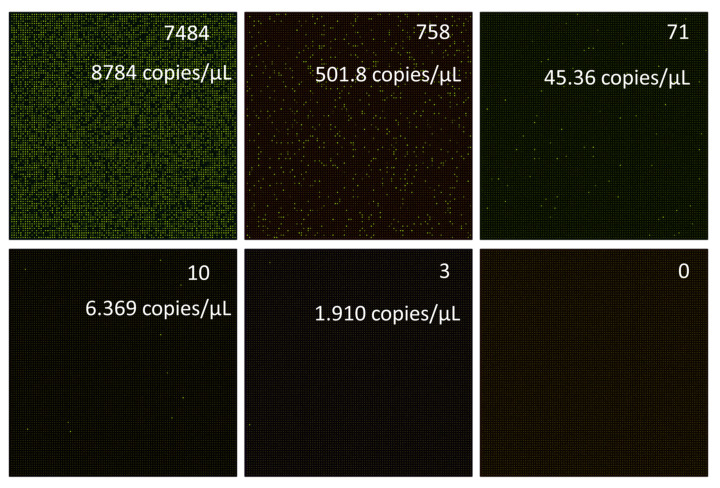
Experimental results of *E. coli* DNA templates with five-fold of dilutions.

On the other hand, we diluted the DNA templates of *E. coli* and conducted a quantitative analysis under concentration gradients. As shown in Figure 5, the first PCR test (with *E. coli* primers and in the HEX channel) was conducted using the initial DNA template extracted from the beef powder. It can be seen that there were 7484/10,000 positive chambers detected in the nested digital PCR test. Based on the calibration equation of the Poisson distribution, the calculated DNA concentration of the *E. coli* pathogen in the beef was 8784 copies/μL. After a 0.1× dilution, the calculated *E. coli* DNA decreased to 501.8 copies/μL. Moreover, the calculated *E. coli* DNA concentration was only 1.910 copies/μL for the 0.0001× dilution of the food samples. For more diluted food samples, the chip gave negative results, probably because the 100 × 100 nested digital PCR chip does not allow us to detect such a low limit of detection. 

### 3.4. Foodborne Pathogen Detection Tests of Real Foods 

To examine the performance of the photothermal nested digital PCR system, we used raw milk and beef as the food samples to conduct a multiplexing pathogen detection test. As shown in Table 2, taking the raw milk as an example, 11 CFU/25 mL of *E. coli* was detected from the fresh milk using the traditional pathogen culture method. Also, the pathogen concentrations of *L. monocytogenes*, *S. aureus*, and *Salmonella* were 36 CFU/25 mL, 20 CFU/25 mL, and 17 CFU/25 mL, respectively. As expected, for the raw milk that was kept at room temperature for 48 h, the pathogen concentration increased significantly. Among them, the content of *L. monocytogenes* even rose as high as 10,862 copies/μL. Thus, it is not recommended to use milk that is untreated or overdue.

### 3.5. Future Work and Limitations 

To note, it would take no less than 24 h (even up to 4~7 days, according to the protocols in the national standard of China, i.e., GB 4789.10-2016, GB 4789.4-2016) for pathogen culture operations to occur. In this case, it would take several hours to extract the DNA of foodborne pathogens. For the proposed photothermal-based nested digital PCR system, it only takes no more than 10 min to complete a nucleic acid amplification procedure. Moreover, image processing would take around 10 min. To sum up, the photothermal effect speeds up the multiplexing foodborne pathogen detection significantly. Our ongoing work aims to integrate the nucleic acid extraction function into the microfluidic chip. With the rapid DNA extraction and PCR reagent preprocessing operations, it is possible to perform ultra-fast multiple foodborne pathogen detection within 30 min.

Moreover, it is recommended to use some other nanomaterials (such as the two-dimensional transition metal dichalcogenide [19,20] or gelatin gold nanorods [21]) to improve the thermal conductivity of the PCR reagents. As alternative functional materials, the nanoflakes or nanorods are expected to have excellent photothermal ability to take an ultra-fast digital PCR test for foodborne pathogen detection or other related applications.

Although the nested digital PCR allows us to identify the absolute quantitative copy numbers of target DNA, there are still several drawbacks of using the PCR-based pathogen detection itself, such as the following: (1) The PCR methods are specialized for pathogen recognition but cannot determine the viability or pathogenicity of any pathogens. For example, the food with a high concentration of *E. coli* may be safe as a food if the pathogen has been destroyed. (2) Using multiplexing for foodborne pathogen detection requires intricate primer/probe design and the optical design of different fluorescence channels. (3) The cost of the PCR system for foodborne pathogens is a big concern for consumers. According to the design of our photothermal digital PCR system, the total cost of all the essential hardware (including the light source, the micro controller, the temperature sensors, the microscopic module, etc.) is around USD 4500, which is slightly more expensive than the equipment that is currently used for the detection of foodborne pathogens. Thus, the development of portable and cost-effective multiplexing foodborne pathogen detection methods is still a challenge. 

## 4. Conclusions

In summary, we proposed a photothermal effect-induced rapid digital PCR system using a microfluidic chip with 100 × 100 nested array. The key elements of the system are the photothermal gold film on the bottom of the chambers and the gold nanoparticles used to improve the thermal conductivity of the PCR reagents. Using the photothermal digital PCR system, we demonstrated rapid nucleic acid amplification within 7 min. The multiplexing fluorescence channels were used for the microscopic imaging of the nested micro chambers. Four kinds of foodborne pathogens (Escherichia coli, Listeria monocytogenes, Staphylococcus aureus, and *Salmonella*) were well distinguished in food samples. The whole system could distinguish those pathogens within 20 min (including thermal cycling and image processing, but the nucleic acid extraction time was not included), showing good potential for the rapid and multiplexing detection of foodborne pathogens.

## Figures and Tables

**Table 1:** Primers and probe channels for multiple foodborne pathogens.

Pathogens	Forward Primer Sequence	Reverse Primer Sequence	Channel
*E. coli*	AGGTGACACTAGAATA TTCGATGAGTTATCTGCAAGGTGA	GTACGACTCACTATAGGGA TAAAGATGTTTTTCACACTTATTGG	HEX
*L. monocytogenes*	AGGTGACACTAGAATA GGGAAATCTGAGGTGAT	GTACGACTCACTATAGGGA GTTTGTTGTATAGGCAATGGG	FAM
*S. aureus*	AGGTGACACTAGAATA GCGATTGATGGTGATACGG	GTACGACTCACTATAGGGA GCCAAGCTTGACGAACTA	ROX
*Salmonella*	AGGTGACACTAGAATA AAATCGTGCAGTGGCTTA	GTACGACTCACTATAGGGA AAGGCGCGGTCTTTACCATC	CY5

**Table 2:** Experimental results of four kinds of foodborne pathogens in milk.

Pathogens	Raw Milk (~0.5 h)	Milk after 48 h
*E. coli*	dPCR: 4 copies/μL pathogen culture: 11 CFU/25 mL	dPCR: 4862 copies/μL pathogen culture: 121 CFU/25 mL
*L. monocytogenes*	dPCR: 12 copies/μL pathogen culture: 36 CFU/25 mL	dPCR: 10,862 copies/μL pathogen culture: 1426 CFU/25 mL
*S. aureus*	dPCR: 3 copies/μL pathogen culture: 20 CFU/25 mL	dPCR: 5042 copies/μL pathogen culture: 179 CFU/25 mL
*Salmonella*	dPCR: 2 copies/μL pathogen culture: 17 CFU/25 mL	dPCR: 4923 copies/μL pathogen culture: 192 CFU/25 mL

## Data Availability

Data are available from corresponding authors by reasonable requests.
